# Relationship Between Cognition and Gait at 2- and 12-Months Post-Traumatic Brain Injury

**DOI:** 10.3389/fresc.2021.726452

**Published:** 2021-11-26

**Authors:** Veronica Vuong, Kara K. Patterson, Lauren Patricia Cole, Tara Lynn Henechowicz, Conor Sheridan, Robin E. A. Green, Michael H. Thaut

**Affiliations:** ^1^Music and Health Science Research Collaboratory, Faculty of Music, University of Toronto, Toronto, ON, Canada; ^2^Institute of Medical Science, Temerty Faculty of Medicine, University of Toronto, Toronto, ON, Canada; ^3^Rotman Research Institute, Baycrest Health Sciences, Toronto, ON, Canada; ^4^Knowledge, Innovation, Talent, Everywhere (KITE) Research Institute, University Health Network, Toronto, ON, Canada; ^5^Department of Physical Therapy, Temerty Faculty of Medicine, University of Toronto, Toronto, ON, Canada; ^6^Rehabilitation Sciences Institute, Temerty Faculty of Medicine, University of Toronto, Toronto, ON, Canada

**Keywords:** cognitive rehabilitation, motor rehabilitation, traumatic brain injury, recovery, rehabilitation

## Abstract

**Background:** A common and debilitating challenge experienced by people with TBI is gait-associated mobility impairment and persisting cognitive impairments. Cognitive and physical impairments are often addressed independently during rehabilitation, however, increasing evidence links cognitive and motor processes more closely.

**Objectives:** (1) To determine if correlations exist between measures of cognitive and gait recovery, post-TBI. (2) To investigate the predictive power of cognition at 2-months on gait outcomes at 12-months post-TBI.

**Methods:** In this secondary, longitudinal study of cognitive and neural recovery, data from 93 participants admitted to an inpatient neurorehabilitation program were analyzed. Spatiotemporal gait variables [velocity, step time variability (STV), step length variability (SLV)] were collected along with cognitive variables [Trail Making Test-B (TMT-B), Digit Span-Forward (DS-F)]. Spearman's correlation coefficients were calculated between gait and cognitive variables. Multilinear and step wise regression analyses were calculated to determine predictive value of cognitive variables at 2-months on gait performance at 12-months-post TBI.

**Results:** At 2-months post-injury, TMT-B was significantly correlated with gait velocity and STV; and DS-F was significantly correlated with velocity. At 12-months post-injury, TMT-B and DS-F was still significant correlated with velocity. TMT-B at 2-months was correlated with SLV and STV at 12-months; and DS-F correlated significantly with velocity. Regression models showed TMT-B at 2-months predicting STV, SLV, and velocity at 12-months.

**Conclusions:** Significant associations and predictions between physical and cognitive recovery post-TBI were observed in this study. Future directions may consider a “neural internetwork” model as a salient rehabilitation approach in TBI that integrates physical and cognitive functions.

## Introduction

A traumatic brain injury (TBI) refers to an external force to the head resulting in structural and/or functional changes to the brain; these are typically associated with acute alterations to consciousness and then transient or persisting cognitive, motor, mood and/or somatic symptoms thereafter (1, 2). A TBI can vary in presentation, depending on severity and region(s) affected (2). TBI is currently the leading cause of death in individuals under the age of 50 in Canada and the United States (2). The prevalence of TBI is increasing, largely due to the increased survival rate of motor vehicle accidents, which is in turn coupled with higher persisting physical and cognitive dysfunction even post-therapy (3).

It is estimated that over three million people with TBI in the United States are living with a significant long-term disability (2, 4, 5). Persisting impairments associated with disability include cognitive deficits (1) and neuromotor impairments (6), such as balance (7). These disabilities adversely affect the livelihood of people with TBI, resulting in loss of productivity. Finkelstein et al. (8) found that after being hospitalized, 1 out of 5 Americans with TBI did not return to work 1 year-post-injury, due to a disability, amounting to an estimated $51.2 billion in total lifetime productivity loss. TBI has become a global public health concern due to the high prevalence of lasting health challenges and is predicted to remain the leading cause of disability stemming from a neurologic disease, until at least 2031 (1, 9).

A common and debilitating challenge experienced by TBI survivors is gait-associated mobility impairment (10, 11). For instance, gait analysis has shown that compared to healthy controls, people with TBI walk slower due to reduced step length and stride length (10, 11). In addition, individuals often report instability during ambulation, revealing deficits in balance (12). As a direct result, people with TBI exhibit increased step-to-step variability (13), may walk slower (11, 12), and report difficulty navigating their environment (10, 12). Yet, despite the existence of strong evidence for persistent motor deficits after TBI when compared to other neurological populations such as Parkinson's disease and stroke, motor impairments have received less attention within the TBI population (14). Mobility challenges, most notably abnormal gait function, have been linked to decreased community engagement (7, 13) and self-rated quality of life, post-TBI (15).

In addition to gait dysfunction, people with TBI exhibit persisting cognitive impairments such as attention, concentration, learning, memory, and executive functioning deficits (16). Executive function can be defined as a group of related, yet distinct, cognitive processes that modulate information from the prefrontal cortex to produce intentional, goal-directed behavior. Models of executive function [e.g., Miyake and Friedman's model (17)] generally include three foundational components: inhibition of pre-potent responses, monitoring and updating, and mental shifting. From these components, basic cognitive processes such as attention control, cognitive inhibition, cognitive flexibility, and working memory, are derived. As these integrative functions are imperative for effective goal directed actions, they play a vital role in independently managing work, social relationships, activities of daily living, as well as re-integrating into the community, post-TBI.

Functionally, cognitive and physical impairments are often addressed independently during rehabilitation, for example by therapies that discretely focus on one (e.g., physiotherapy) or the other (e.g., speech language therapy; neuropsychology) (11). However, increasing evidence has shown that cognitive and motor processes are closely linked (11). Motor function is mediated by complex cortical, subcortical, and brainstem networks that communicate with each other and project signals *via* the reticulospinal pathway to the spinal cord, resulting in coordinated movement (18, 19). TBI can disrupt one or several pathways implicated in cognitive functioning, which may impede motor functioning, such as gait (20–22). For example, impaired executive function is associated with slower walking speed (20), increased incidence of falls (23, 24), and increased stride time variability (25). Furthermore, dual-tasking has demonstrated the interplay between executive function and gait, both of which are commonly affected after TBI (20, 26).

Although functional outcomes following TBI have previously been predicted by demographic variables and severity of brain injury, they have produced mixed results (27). Alternatively, there has been a new effort to focus research on cognitive sequela due to TBI. Based on the evidence that cognitive function is associated with motor impairments, especially in locomotion, the question arises if cognitive outcomes can also become predictors of motor outcomes. For example, emerging evidence shows associations between cognition and motor recovery in stroke, most pronounced for executive function (28). However, such potential connections have not yet been investigated for TBI. Therefore, this study is a novel investigation that seeks to close the existing knowledge gap to improve outcomes for successful TBI rehabilitation. If associations or predictions are found, this may present a platform for future research to integrate both cognitive and motor domains in rehabilitation exercises. Previous studies involving persons with TBI have examined cognitive and motor recovery separately. Therefore, this investigation presents a novel approach in analyzing the long-term relationship between cognitive domains, particularly executive function, and gait as one of the most critical motor outcomes during post-trauma recovery.

We sought to investigate if correlations may exist between measures of executive function and gait recovery, post-TBI. As our first hypothesis, we predicted that significant correlations would emerge in early recovery and would be maintained longer term between cognition and gait performance. Additionally, we sought to investigate the predictive power of executive functioning at 2-months post-TBI on gait outcomes at 12-months post-TBI. As our second hypothesis, we predicted that executive function in early recovery stages is a significant predictor for long-term gait outcomes.

## Materials and Methods

### Participants

The data of participants who were admitted to the inpatient neurorehabilitation program of a major urban Canadian hospital and who took part in a parent, longitudinal study of cognitive and neural recovery [“The Toronto Rehab TBI Recovery Study” (29)] were analyzed for this statistical follow up investigation with all demographic and diagnostic data additionally available through the parent study.

A total of 93 individuals qualified for the current study by meeting the following inclusion criteria: [1] acute care diagnosis of TBI; [2] severity as indicated by length of post-traumatic amnesia (LPTA) of >1 h and/or Glasgow Coma Scale (GCS) score of <12 either at their emergency admission or the scene of the accident and /or positive computed tomography or MRI findings; [3] age between 18 and 80 years; [4] ability to comprehend and follow simple communication in English based on speech language pathology intake assessment; [5] informed consent provided by the participant or legal decision-maker.

Exclusion criteria included: [1] diagnosis of an additional independent neurological condition such as Alzheimer's disease, Parkinson's disease, multiple sclerosis, Huntington's disease, lupus, or stroke; [2] history of psychiatric disorder; [3] the etiology of the TBI resulting from a pre-existing or acute neurologic condition, such as a fall caused by a stroke; [4] physical assistance required to complete gait tests; [5] orthopedic injuries affecting both lower extremities; [6] failure to emerge from LPTA by 6 weeks post injury, as measured by the Galveston Orientation Amnesia Test.

Mean participant age was 41 ± 17 with 26 women in the group. No significant differences were found for age using a two-way ANOVA (*F* = 0.11; *p* = 0.89). On average, participants had sustained a moderate to severe brain injury (mean lowest recorded score/GCS 6.97 ± 3.59; severe <8 = 69.8 %; moderate 9–12 = 18,7%; mild 13–15 = 11.5%) which resulted in a mean acute care length of stay of 33 ± 18.6 days with rehabilitation starting after physical stabilization of the patient (LPTA: moderate 1–24 h = 4.1%; severe 1–7 d = 24.1%; very severe 1–4 w = 54.4%; extremely severe 4–6 w = 17.4%). Average number of therapy hours per week was 7.07 ± 4.68. The etiology was reported as follows: motor vehicle collision (70.8%), fall (12.5%), assault (12.5%), and sports injury (4.2%). Pre-morbid IQ of participants was 100.4 ± 12.51 [Wechsler Test of Adult Reading (30) or North American Adult Reading Test-Verbal IQ (31)]. Our sample was typical for moderate to severe TBI, predominantly male, with an estimated average pre-morbid IQ, and the majority of injuries due to motor vehicle accident.

### Measures

#### Gait Variables

All spatiotemporal gait variables were collected and calculated using a GaitRite pressure-sensitive mat system. Spatiotemporal gait measures collected by a pressure-sensitive system have been shown to be valid and reliable in both inpatient (32) and outpatient (33) populations post-TBI. Variables were collected at each timepoint (2- and 12-months post-admission) under the following two conditions: self-paced (SP) where participants were asked to walk at a self-selected comfortable walking speed; maximum-paced (MP) where participants were asked to walk as fast as possible while maintaining safety.

Each participant walked independently across the pressure-sensitive mat for a total of 18 footfalls. Participants began walking 2.5 m before the beginning of the mat and continued for 2.5 meters following the end of the mat to account for acceleration and deceleration associated with starting and stopping. At the time of collection, a research assistant cleaned the data by removing incomplete footfalls (at the beginning or end of the mat) and any other abnormalities or artifacts detected by the system. The GaitRite computer system calculated all spatiotemporal variables following data cleaning.

We used gait velocity (measured in cm/s) as a global measure of gait function. Velocity is commonly considered a critical and sensitive measure in assessing functional gait and overall health sometimes referred to as the “6^th^ vital sign” (34, 35). Furthermore, for individuals with reduced mobility following TBI, walking speed may be an important measure of successful future community integration (35). The coefficient of variation of step length variability (SLV) and coefficient of variation of step time variability (STV) were used to measure gait variability indicating the ratio of the standard deviation to the mean. SLV and STV were selected as critical factors for timing and pattern generation of walking movements. Consistent SLV and STV across gait cycles are also important for stability of gait and reduction of risk of falling (36).

A custom MATLAB program was used to calculate SLV and STV as the coefficient of variation from the GaitRite output of footfall location and timing.

#### Neuropsychological Test Variables

We used Trail Making Test-B (TMT-B) (37) total time score as a measure of core components of executive function and the Digit Span-Forward (DS-F) (38) as a measure of simple auditory-verbal attention and capacity.

The TMT-B is a timed, visuomotor task that is frequently used in clinical evaluations because of its sensitivity to impairment (37). It requires participants to alternately connect letters and numbers in numerical and alphabetical sequence as quickly as possible. Poor performance has been associated with reduced mental shifting, cognitive flexibility and working memory (39).

The DS-F requires the participant to repeat sequences of numbers that increase in length (and therefore difficulty) that are read out loud.

TMT-B was selected for this analysis as it has shown to be related to physical functioning (40) and to also account for the largest proportion of variance on the instrumental activities of daily living (IADL) test (41). Additionally, executive function impairments have been noted to be significant predictors of falls in TBI rehabilitation, attributing this finding to reduced self-awareness, risk assessment skills, attentional challenges, and impulsivity (23). We chose a measure of attention due to the strong evidence that control of attention is a critical ability during walking (20).

### Design

The current study is a secondary, longitudinal follow up statistical analysis of the above referenced parent study using selected variables to investigate potential relationships between cognition and motor function after TBI.

### Data Analysis

First, repeated measures ANOVA was calculated for differences between 2- and 12-months as well for SP vs. MP performance in gait velocity (cm/s), cadence (steps/m), step time (s), and step length (cm).

To test our first hypothesis, Spearman's correlation coefficients were calculated for each combination of one of the three gait variables (velocity, step length, and step time variability) and one of the two cognitive variables (TMT-B, DS-F): (1) concurrent time point analysis at 2- and 12-months post-TBI; (2) cognition variables at 2-months and gait variables at 12-months. All correlations were completed in the statistical package R. The Holm multiple test procedure was employed for all analyses (42). Adjusted *p*-values are reported.

To test our second hypothesis, multilinear and step wise regression analyses were calculated to determine if cognitive variables at 2-months can predict gait performance at 12-months.

All Multiple Linear Regression Analyses were computed using the “rms” package in R (43). Six Multiple Linear Regression Models were fitted to test whether 2-month cognition variables, TMT-B and DS-F, predict each 12-month gait variable at SP and MP. The six 12-month gait outcome variables were 12-month SP SLV, 12-month MP SLV, 12-month SP STV, 12-month MP STV, 12-month MP Velocity, and 2-month MP Velocity. Thus, the equation for each fitted model is: y = β0 + β1 (2-month TMT-B) + β2 (2-month DS-F). Additionally, an ANOVA of the fitted regression model was conducted for each 12-month gait variable.

## Results

### Descriptive Statistics

Descriptive cognitive statistics are given in Table 1. Descriptive gait statistics are given in Table 2.

**Table 1:** Descriptive statistics for cognitive variables.

**Months**	**Neuropsychological test variable**	**Mean**	**SD**
2-Months	TMT-B	100.4 s	35.4
12-Months	TMT-B	85.7 s	28.3
2-Months	DS-F	9.9 correct trials	2.7
12-Months	DS-F	10.1 correct trials	2.7

*DS-F, Digit Span-Forward; TMT-B, Trail Making Test-B*.

**Table 2:** Descriptive statistics for gait variables.

**Months**	**Gait variable**	**Condition**	**Mean**	**SD**
2-Months	Velocity	MP	180.2 cm/s	42.7
2-Months	Velocity	SP	113.3 cm/s	30.2
12-Months	Velocity	MP	194.8 cm/s	33.7
12-Months	Velocity	SP	116.8 cm/s	25.5

*Velocity = cm/s; MP, max-paced; SP, self-paced*.

There was a significant main effect for time. Significant increases for velocity were found between 2- and 12-months in the SP condition (*p* = 0.0022) and in the MP condition (*p* = 0.02).

### Correlations

#### Concurrent Timepoint Analysis—Spearman's Correlations Between Cognition and Gait at 2-Months Post

At 2-months post-injury, test performance on the TMT-B was significantly correlated with gait velocity as well as step time variability in both SP and MP conditions. The DS-F test was only correlated significantly with the MP velocity condition (Table 3).

**Table 3:** Correlations between cognition and gait 2-months post-admittance to the traumatic brain injury (TBI) recovery program.

**Condition**	**Gait variable**	**Cognitive variable**	**Correlation coefficient**	***P*** **-value**
MP	Velocity	TMT-B	−0.28	0.0096*
SP	Velocity	TMT-B	−0.33	0.0028*
MP	Velocity	DS-F	0.26	0.0199*
SP	Velocity	DS-F	0.16	0.1393
MP	SLV	TMT-B	0.1	0.5225
SP	SLV	TMT-B	0.17	0.2531
MP	STV	TMT-B	0.32	0.0326*
SP	STV	TMT-B	0.39	0.0073*
MP	SLV	DS-F	−0.02	0.9185
SP	SLV	DS-F	−0.13	0.4113
MP	STV	DS-F	−0.14	0.363
SP	STV	DS-F	−0.06	0.6864

*MP, max-paced; SP, self-paced; SLV, step length variation; STV, step time variation; TMT-B, Trail Making Test-B; DS-F, Digit Span-Forward*.

* * means Significant p-value = 0.05*.

#### Concurrent Timepoint Analysis—Spearman's Correlations Between Cognition and Gait at 12-Months Post-admittance

At 12-months post-injury, the number of significant correlations between cognition and gait had decreased. Both the TMT-B and DS-F were still correlated with SP velocity, but other associated performance linkages between gait and cognition had disappeared (Table 4).

**Table 4:** Correlations between cognition and gait 12-months post-admittance to the traumatic brain injury (TBI) recovery program.

**Condition**	**Gait variable**	**Cognitive variable**	**Correlation coefficient**	***P*** **-value**
MP	Velocity	TMT-B	−0.2	0.1499
SP	Velocity	TMT-B	−0.37	0.007*
MP	Velocity	DS-F	0.22	0.1029
SP	Velocity	DS-F	0.34	0.0115*
MP	SLV	TMT-B	0.19	0.2848
SP	SLV	TMT-B	0.33	0.0572
MP	STV	TMT-B	0.23	0.1839
SP	STV	TMT-B	0.32	0.0649
MP	SLV	DS-F	−0.09	0.5989
SP	SLV	DS-F	−0.1	0.57
MP	STV	DS-F	−0.15	0.4039
SP	STV	DS-F	−0.12	0.5028

*MP, max-paced; SP, self-paced; SLV, step length variation; STV, step time variation; TMT-B, Trail Making Test-B; DS-F, Digit Span-Forward*.

* *means Significant p-value = 0.05*

#### Spearman's Correlations Between Cognition 2-Months and Gait 12-Months

When correlating cognition at 2-months post-injury with gait recovery at 12-months post-injury, new significant findings emerged showing that early and higher performance on TMT-B was associated with improvements in gait variability for step length as well as step time. Higher DS-F scores at 2-months post-injury maintained a significant association with higher gait velocity even across a 10-month time span between testing, however, for SP only (Table 5).

**Table 5:** Correlations between cognition at 2-months post-injury and gait at 12-months post-injury to the traumatic brain injury (TBI) recovery program.

**Condition**	**Cognitive variable at 2 months post-injury**	**Gait variable at 12 months post-injury**	**Correlation coefficient**	***P*** **-value**
SP	TMT-B	Velocity	−0.18	0.2214
MP	TMT-B	Velocity	−0.1	0.4788
SP	TMT-B	SLV	0.51	0.0076*
MP	TMT-B	SLV	0.04	0.8324
SP	TMT-B	STV	0.53	0.0005*
MP	TMT-B	STV	0.11	0.5869
SP	DS-F	Velocity	0.44	0.0021*
MP	DS-F	Velocity	0.29	0.045*
SP	DS-F	SLV	−0.31	0.1179
MP	DS-F	SLV	−0.07	0.726
SP	DS-F	STV	−0.15	0.4794
MP	DS-F	STV	−0.23	0.2606

*MP, max-paced; SP, self-paced; SLV, step length variation; STV, step time variation; TMT-B, Trail Making Test-B; DS-F, Digit Span-Forward*.

* *means Significant p-value = 0.05*

### Regression

Of the six multiple linear regression models, 2-month post-injury TMT-B was significantly predictive of 12-month post-injury gait in the models for 12-month SP SLV (β = 0.0113, SE = 0.0051, and *p* = 0.0317), 12-month SP STV (β = 0.0103, SE = 0.0046, and *p* = 0.0317), and MP Velocity (β = −0.3320, SE = 0.1081, and *p* = 0.0039; see Table 6 for the Discrimination Indices and Regression Coefficients for each model). DS-F was not a significant predictor in any of the models.

**Table 6:** Identifying 2-month cognition predictors of 12-month gait in people post-traumatic brain injury (TBI) from multiple linear regression outputs.

**Model of dependent gait variable**	**Discrimination indexes**		**Independent cognitive variable**	**Regression coefficients**			
	**Model *R*^2^**	**Model *R*^2^ adjusted**		**β**	**SE**	**T**	**Regression sig. *p***
12-month SP STV	0.117	0.071	2-month TMT-B	0.0103	1.2157	2.21	0.0331*
			2-month DS-F	0.0458	0.0945	0.48	0.6305
12-month MP STV	0.043	−0.006	2-month TMT-B	0.0038	0.0064	0.59	0.5582
			2-month DS-F	−0.1068	0.1292	−0.83	0.4133
12-month SP SLV	0.129	0.084	2-month TMT-B	0.0113	0.0051	2.23	0.0317*
			2-month DS-F	0.0150	0.1031	0.15	0.8854
12-month MP SLV	0.038	−0.011	2-month TMT-B	0.0051	0.0044	1.16	0.2543
			2-month DS-F	0.0081	0.0898	0.09	0.9288
12-month SP Velocity	0.071	0.024	2-month TMT-B	−0.0908	0.0843	−1.08	0.2881
			2-month DS-F	1.3128	1.7135	0.77	0.4482
12-month MP Velocity	0.220	0.180	2-month TMT-B	−0.3320	0.1081	−3.07	0.0039*
			2-month DS-F	−0.4022	2.1968	−0.18	0.8557

* *P-values are significant at the alpha = 0.05 significance level*.

*MP, max-paced; SP, self-paced; SLV, step length variation; STV, step time variation; TMT-B, Trail Making Test-B; DS-F, Digit Span-Forward*.

**Figure 1:**
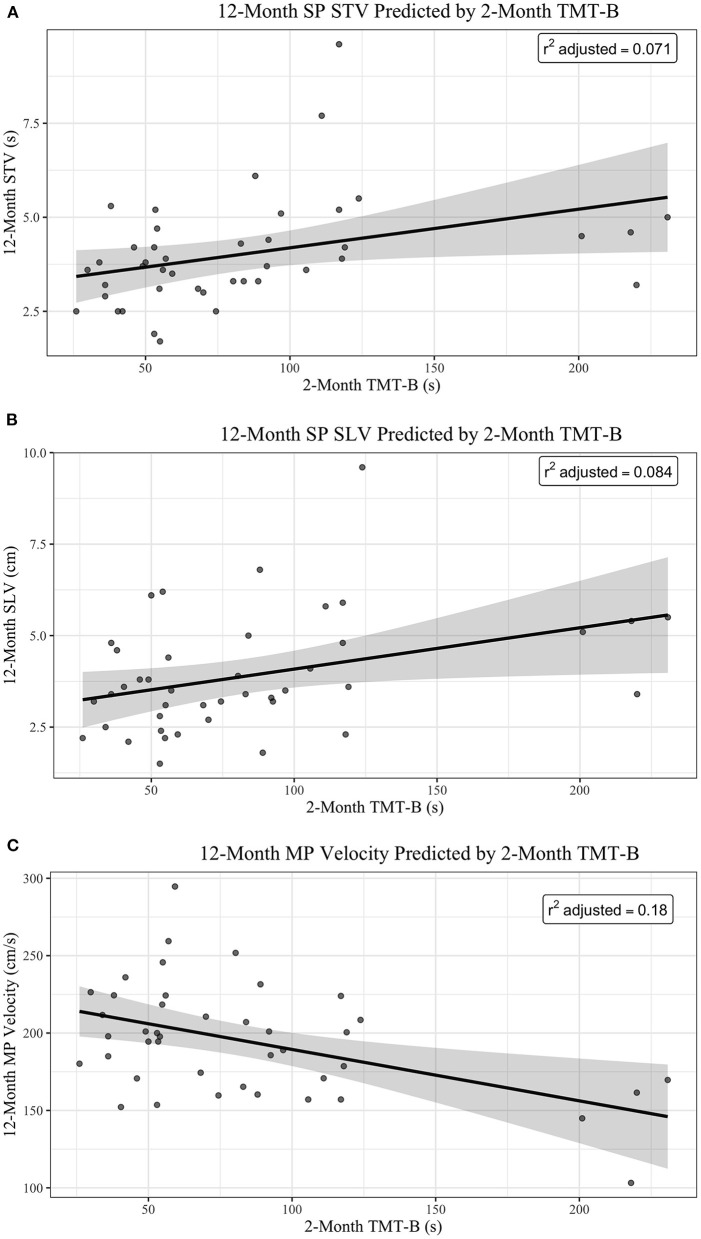
**(A)** 12-month SP STV predicted by 2-month TMT-B. **(B)** 12-month SP SLV predicted by 2-month TMT-B. **(C)** 12-month MP velocity predicted by 2-month TMT-B.

For every 30 s increase in time to complete TMT-B at 2-months post-injury, 12-month SP STV increases by 0.339150 s (95% CI of 0.031365–0.64693), when adjusting for DS-F (see Figure 1A). For every 30 s increase in time to complete 2-month TMT-B, 12-month SP SLV increases by 0.3084 cm (95% CI of 0.026253–0.59054), when adjusting for DS-F (see Figure 1B). For every 30 s increase in time to complete 2-month post-injury TMT-B, 12-month post-injury MP Velocity decreases by 9.9613 cm/s (95% CI of −16.518 to −3.4044), when adjusting for DS-F (see Figure 1C). However, our adjusted *R*^2^ values, measuring goodness of fit, are low, indicating the percentage of the variances in the dependent variables that the independent variables explain collectively. This suggests that our models are underfitting the variability in the data, limiting the predictive precision of the relationships.

To assure that our results were not leveraged by outlier data points, we first conducted a sensitivity analysis with dfbetas and threshold set at 0.3 determined by 2/sqrt(*n*) to identify the most influential observations and then removed them in an updated model analysis. We considered this as an appropriate first step because it is computationally driven and not an arbitrary decision based on visual inspection. TMT-B was still a significant predictor of STV (*p* = 0.033 and *R*^2^ adjusted changed from 0.071 in the original model to 0.172. Similar results were found for SLV (*p* = 0.0243; *R*^2^ from 0.084 to 0.111) and velocity (*p* = 0.0057; *R*^2^ from 0.18 to 0.16). In a second step, we finally removed four outlier observations with TMT-B > 150 s. TMT-B remained a significant predictor for STV (*p* = 0.0008; *R*^2^adj = 0.237) and SLV (*p* = 0.0244; *R*^2^adj = 0.092).

When controlling for age and gender, the main effects for 2-month TMT-B on 12-month SP STV (β = 0.0118, SE = 0.0054, and *p* = 0.0357), 12-month SP SLV (β = 0.0130, SE = 0.0059, and *p* = 0.0337), and 12-month MP Velocity (β = −0.3682, SE = 0.1250, and *p* = 0.0055) remained significant. The main effects of age and gender were not significant in any of the three models.

Figure 1 shows plots of the main effect of 2-month TMT-B (sec) on (A) 12-Month SP, (B) 12-Month SP SLV, and (C) 12-Month MP Velocity. The regression lines are shown in black with 95% Confidence Interval (in gray). The *R*^2^ adjusted is reported in the top right corner of each plot.

When controlling for age and gender, the main effects for 2-month TMT-B on 12-month SP STV (β = 0.0118, SE = 0.0054, and *p* = 0.0357), 12-month SP SLV (β = 0.0130, SE = 0.0059, and *p* = 0.0337), and 12-month MP Velocity (β = −0.3682, SE = 0.1250, and *p* = 0.0055) remained significant. The main effects of age and gender were not significant in any of the three models.

## Discussion

Traditionally, physical and cognitive impairments post-TBI have been addressed separately, and therefore, their rehabilitation processes have occurred independently (11). There is increasing evidence suggesting that physical processes such as ambulation require higher order cognitive inputs, including executive function and attention (11). Therefore, it is imperative to understand the relationship between cognitive function and gait post-TBI in order to create effective rehabilitation protocols for both cognitive and physical function. This study presents new data to elucidate the relationship between cognitive functioning and gait post-TBI with the aim to provide preliminary evidence for the creation of effective rehabilitation protocols that addresses cognitive and physical function in an integrated fashion. The current investigation shows evidence for pre-rehabilitation and post-rehabilitation correlations and predictions that exist between measures of gait variability and gait velocity and executive function and attention. The results offer several key findings.

First, in the early stages of recovery, levels of cognitive function and gait function seem closely correlated. TMT-B and DS-F scores significantly correlated with both SP and MP gait speed measures; and reductions in temporal stride variability, the latter being a key indicator of gait control.

Second, long-range correlations persist between cognition and gait at 2- and 12-months, respectively. Higher executive function status in early recovery is associated with long-term improvements in gait variability and higher attention function in early recovery and is associated with long-term improvements in gait velocity.

Finally, our regression analysis shows scores on the TMT-B as a significant predictor of spatial and temporal gait control variability as well as gait speed, even when controlled for age, gender, and influence of outlier observations. Although a predictive relationship was found between the independent variables, the low adjusted *R*^2^ values require a cautionary interpretation of the limited precision of the predictions.

Previous studies have shown connections between executive and attention function and motor control, including gait functions (3) [e.g., (20, 22, 44–46)]. While the connections between cognition and motor function have been mostly investigated in cohorts of healthy elderly persons and persons with stroke and Parkinson's disease, our study for the first time shows similar connections in TBI recovery. Furthermore, we were able to elucidate a predictive role for early positive cognitive recovery, especially for executive function, for improvement of critical kinematic gait variables in long-range gait control after 1 year.

There is a consistent body of research showing the importance of physical ability for maintaining cognitive function. However, our data show that there is also a reversed role for cognitive function predicting long-term motor outcomes. One challenge associated with TBI treatment and rehabilitation is that unlike other neurological conditions, there is not one common mechanism of injury (3). The many ways one can obtain a TBI and present clinically lead to a plethora of diverse symptoms. This study has shown that cognition is predictive of gait, across a spectrum of TBI injuries. This evidence may lead to a more holistic strategy in early TBI rehabilitation to design rehabilitation exercises that integrate appropriate cognitive and motor challenges in re-learning protocols. Early studies examining dual task intervention programs for individuals with acquired brain injury have demonstrated a lack of generalizability (47). Further studies are required to assess the efficacy of concurrent cognitive-motor training regimes.

Our data confirm that at least at a functional and goal-directed level, walking involves executive and attention capabilities, and if those capabilities are better preserved post-injury, they can be important predictors for long-term improvements of walking ability. What mechanisms may account for the associative and predictive relationship? Poor cognition is typically associated with poor rehabilitation outcomes. One of the suggested mechanisms borrows from stroke research as a model for acquired brain injury and postulates that poor cognition alters implicit and explicit learning abilities which are important for recovery in stages of adaptation as well as remediation on an impairment level (19, 48). This mechanism may also underlie the outcomes in our present TBI study. On the neural level, studies have pointed toward possible pathways that link cognition and motor outcomes at alterations in the neurochemical milieu (49). Thus, within the context of shared pathways in neurotransmitter signaling, recovery of cognitive impairment may also provide a more favorable neurochemical milieu for functional recovery of motor control, which in our case would specifically pertain to gait mobility (50). Finally, our model data may suggest that they share representations in the brain, e.g., in tertiary association areas, or share a common network characterized by long-range internetwork connectivity (51).

## Conclusions/Implications

Our study presents novel data in regard to significant associations and predictions, however, with limited model fit, between cognitive and motor recovery in TBI. These associations and predictions have not been previously researched. Therefore, as opposed to traditional approaches, we suggest that further investigations address cognition and motor control in an integrated fashion based on a “neural internetwork” model of rehabilitating impaired brain function.

## Data Availability Statement

The original contributions presented in the study are included in the article/supplementary material, further inquiries can be directed to the corresponding author/s.

## Ethics Statement

The studies involving human participants were reviewed and approved by Toronto Rehabilitation Institute. The patients/participants provided their written informed consent to participate in this study.

## Author Contributions

VV: conceptualization, design/methodology, writing original draft, data collection, and data analysis. KP: conceptualization, design/methodology, and review and editing. LC and TH: data analysis. CS: design/methodology and data collection. RG: review and editing. MT: conceptualization, design/methodology, writing original draft, data analysis, and supervision. All authors contributed to the article and approved the submitted version.

## Conflict of Interest

The authors declare that the research was conducted in the absence of any commercial or financial relationships that could be construed as a potential conflict of interest.

## Publisher's Note

All claims expressed in this article are solely those of the authors and do not necessarily represent those of their affiliated organizations, or those of the publisher, the editors and the reviewers. Any product that may be evaluated in this article, or claim that may be made by its manufacturer, is not guaranteed or endorsed by the publisher.
